# Osmosensitivity of Transient Receptor Potential Vanilloid 1 Is Synergistically Enhanced by Distinct Activating Stimuli Such as Temperature and Protons

**DOI:** 10.1371/journal.pone.0022246

**Published:** 2011-07-14

**Authors:** Eri Nishihara, Takeshi Y. Hiyama, Masaharu Noda

**Affiliations:** 1 Division of Molecular Neurobiology, National Institute for Basic Biology, The Graduate University for Advanced Studies, Okazaki, Aichi, Japan; 2 School of Life Science, The Graduate University for Advanced Studies, Okazaki, Aichi, Japan; Dalhousie University, Canada

## Abstract

In animals, body-fluid osmolality is continuously monitored to keep it within a narrow range around a set point (∼300 mOsm/kg). Transient receptor potential vanilloid 1 (TRPV1), a cation channel, has been implicated in body-fluid homeostasis *in vivo* based on studies with the *TRPV1*-knockout mouse. However, the response of TRPV1 to hypertonic stimuli has not been demonstrated with heterologous expression systems so far, despite intense efforts by several groups. Thus, the molecular entity of the hypertonic sensor *in vivo* still remains controversial. Here we found that the full-length form of TRPV1 is sensitive to an osmotic increase exclusively at around body temperature using HEK293 cells stably expressing rat TRPV1. At an ambient temperature of 24°C, a slight increase in the intracellular calcium concentration ([Ca^2+^]_i_) was rarely observed in response to hypertonic stimuli. However, the magnitude of the osmosensitive response markedly increased with temperature, peaking at around 36°C. Importantly, the response at 36°C showed a robust increase over a hypertonic range, but a small decrease over a hypotonic range. A TRPV1 antagonist, capsazepine, and a nonspecific TRP channel inhibitor, ruthenium red, completely blocked the increase in [Ca^2+^]_i_. These results endorse the view that the full-length form of TRPV1 is able to function as a sensor of hypertonic stimuli *in vivo*. Furthermore, we found that protons and capsaicin likewise synergistically potentiated the response of TRPV1 to hypertonic stimuli. Of note, HgCl_2_, which blocks aquaporins and inhibits cell-volume changes, significantly reduced the osmosensitive response. Our findings thus indicate that TRPV1 integrates multiple different types of activating stimuli, and that TRPV1 is sensitive to hypertonic stimuli under physiologically relevant conditions.

## Introduction

Mammals have a set of homeostatic mechanisms that work together to maintain body-fluid osmolality at near 300 mOsm/kg through the intake or excretion of water and salt [1], [2]. This homeostatic osmoregulation is vital, because changes in cell volume caused by severe hypertonicity or hypotonicity can lead to the irreversible damage of organs and cause lethal neurological trauma [3]–[5]. However, the mechanisms for the detection of these fluctuations have not been fully elucidated.

TRPV1 was suggested to be involved in the detection of hypertonicity based on physiological studies with *TRPV1*-knockout (*TRPV1*-KO) mice [6]. The mice showed pronounced serum hypertonicity under basal conditions and highly compromised vasopressin (VP) production in response to osmotic stimulation *in vivo* [7]. They also showed a significantly attenuated water intake in response to systemic hypertonicity compared with wild-type (WT) controls [8]. However, another group recently claimed that *TRPV1*-KO mice displayed normal thirst responses and central Fos activation during hypernatremia [9]. According to the former group, cells in the supraoptic nucleus (SON) or organum vasculosum of the lamina terminalis (OVLT) of *TRPV1*-KO mice lacked sensitivity to hypertonicity [7], [8]. In this study, a putative N-terminal variant of the TRPV1 channel was postulated as a sensor for hypertonic stress *in vivo* [7]. On the other hand, the osmosensitivity of the full-length form of TRPV1 has not been demonstrated to date by using cell-based expression systems.

As its counterpart, TRPV4 has been reported to be the sensor for the detection of systemic hypotonicity [10]–[12]. A similar discrepancy in results exists for *TRPV4*-KO mice. One group reported an impairment in the stimulation of Fos expression in OVLT neurons, as well as in thirst and VP production, in response to hypertonicity [13]. However, another group detected no difference in water intake, but excessive production of VP in response to hypertonicity [14]. The response of the TRPV4 channel to hypotonic stimuli is sensitive to temperature [10]: Interestingly, the peak sensitivity of the gating of chick and rat TRPV4 was recorded at the core body temperature of the respective animals.

TRPV1 was originally cloned as an endogenous sensor responding to noxious heat (>43°C), in addition to capsaicin and protons [15]. In the present study, we attempted to figure out why the osmosensitivity of the full-length TRPV1 has not been detected so far, by using a heterologous expression system. Here, we demonstrate for the first time that TRPV1 shows clear sensitivity to hypertonic stimuli when ambient temperature is around the mammalian body temperature. Moreover, the osmosensitivity of TRPV1 is potentiated also by other stimuli such as protons and capsaicin.

## Results

### TRPV1-Expressing Cells Show a Temperature-Dependent Response to Hypertonic Stimulation

**Figure 1:**
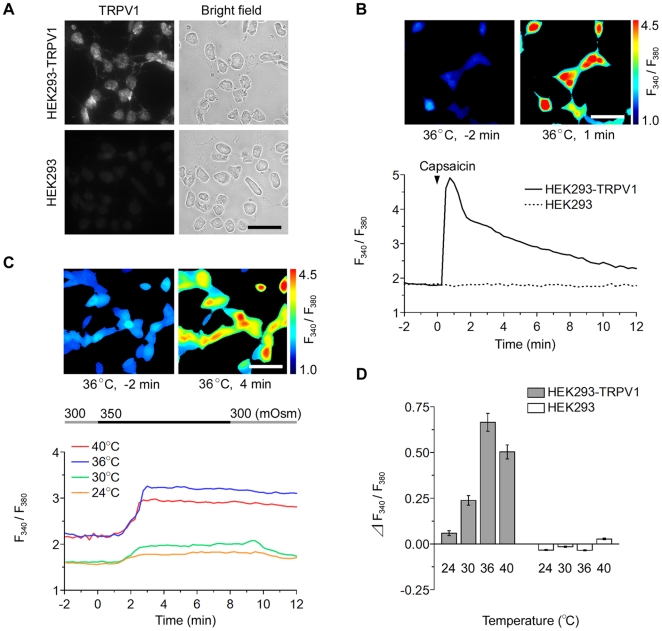
HEK293-TRPV1 cells respond to hypertonic stimuli in a temperature-dependent fashion. (**A**) Immunostaining of HEK293-TRPV1 and HEK293 cells with anti-TRPV1 antibody. Signals for TRPV1 were predominantly observed at the plasma membrane specifically in HEK293-TRPV1 cells. Bar, 40 µm. (**B**) Cell response to capsaicin. Representative pseudo-color images of HEK293-TRPV1 cells at 2 min before (upper left) and 1 min after (upper right) a drop of addition of 1 µM capsaicin to the chamber at 36°C. Elevations of the intracellular calcium concentration ([Ca^2+^]_i_) are shown by an increase in the ratio of F_340_/F_380_ (see color bar). Representative traces of the fluorescence ratio of single cells (lower graph) upon application (arrowhead) of capsaicin; HEK293-TRPV1 (solid line) and control HEK293 (dotted line). Bar, 40 µm. (**C**) Temperature-dependent sensitivity to osmotic stimuli. Representative pseudo-color images of [Ca^2+^]_i_ in HEK293-TRPV1 cells at 2 min before (upper left) and 4 min after (upper right) perfusion with a hypertonic solution (350 mOsm) at 36°C. The temperature was maintained throughout the respective recording. Representative single cell traces of the fluorescence ratio at a temperature of 24, 30, 36, or 40°C (lower graph). The line on the top indicates the timing of the change from 300 mOsm (gray) to 350 mOsm (black). The response of HEK293-TRPV1 cells to hypertonic stimuli increased noticeably with the experimental temperature up to 36°C. Bar, 40 µm. (**D**) Summary of the change in the fluorescence ratio during the perfusion with the hypertonic solution at various temperatures in HEK293-TRPV1 (filled bars) and HEK293 (open bars) cells. Data are differences between fluorescence ratios 2 min before and 4 min after the change of the solution. The maximal sensitivity of the HEK293-TRPV1 cells to the hypertonic stimulation was observed at 36°C. Values are the mean ± SEM. HEK293-TRPV1: *n* = 147 (24°C), *n* = 121 (30°C), *n* = 249 (36°C), *n* = 106 (40°C). HEK293: *n* = 110 (24°C), *n* = 132 (30°C), *n* = 109 (36°C), *n* = 101 (40°C).

First of all, the expression of the full-length form of the TRPV1 channel in the human embryonic kidney (HEK) 293 cells stably expressing rat TRPV1 (HEK293-TRPV1; see Materials and Methods) was verified by RT-PCR using primer sets for rat *TRPV1* mRNA (Fig. S1, HEK293-TRPV1), immunostaining with anti-TRPV1 antibody (Fig. 1*A*, HEK293-TRPV1), and calcium imaging with a TRPV1 agonist, capsaicin (1 µM; Fig. 1*B*, HEK293-TRPV1). Native HEK293 cells were deficient in the endogenous expression of TRPV1, and used for control experiments (Fig. 1*A and B*; HEK293; see also [15]). Changes in the intracellular calcium concentration, [Ca^2+^]_i_, was examined by ratiometric calcium imaging with Fura-2.

To test our idea that the osmosensitivity of the TRPV1 channel is triggered by an increase in ambient temperature, a hypertonic solution (350 mOsm) was applied to HEK293-TRPV1 cells at various temperatures (24, 30, 36, and 40°C) (Fig. 1*C and D*, HEK293-TRPV1). When the extracellular environment was changed from an isotonic (300 mOsm) to hypertonic (350 mOsm) solution at 24°C, [Ca^2+^]_i_ increased only slightly in HEK293-TRPV1 cells, but did not change in HEK293 cells (Fig. 1*C and D*, 24°C). Surprisingly, the sensitivity of HEK293-TRPV1 cells to the osmotic stimulation increased markedly with a rise in temperature (Fig. 1*C and D*, 30, 36, and 40°C): The baseline showed a considerable difference between 30°C and 36°C, partly because of the temperature dependence of fura-2 fluorescence [16]. The sensitivity of the rat TRPV1 was maximal at 36°C, which is close to the normal mammalian body temperature (∼37°C): The [Ca^2+^]_i_ increase (ΔF_340_/F_380_) induced by 350 mOsm was approximately a half of that induced with 1.5 nM capsaicin (see below). In contrast, control HEK293 cells did not respond to the hypertonic stimulus at any temperature. This indicates that TRPV1 is responsible for the osmosensitivity.

### TRPV1-Expressing Cells Show Relatively Small Sensitivity to Hypotonicity

**Figure 2:**
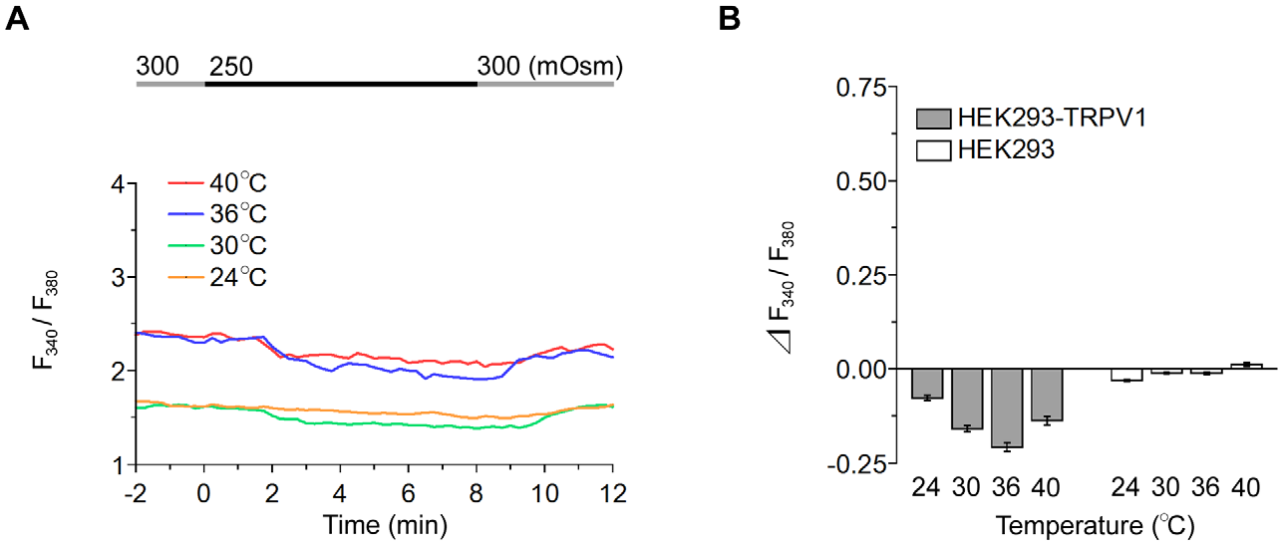
HEK293-TRPV1 cells scarcely respond to hypotonic stimuli in a temperature-dependent fashion. (**A**) Representative single cell traces of the fluorescence ratio during perfusion with a hypotonic solution (250 mOsm) at various temperatures. The temperature was maintained throughout the respective recording. The line on the top indicates the timing of the change in osmolality between 300 mOsm (gray) and 250 mOsm (black). (**B**) Summary of the change in the fluorescence ratio in HEK293-TRPV1 (filled bars) and HEK293 (open bars) cells. Data are differences between fluorescence ratios 2 min before and 4 min after the change of the solution. Values are the mean ± SEM. HEK293-TRPV1: *n* = 147 (24°C), *n* = 145 (30°C), *n* = 105 (36°C), *n* = 108 (40°C). HEK293: *n* = 111 (24°C), *n* = 102 (30°C), *n* = 112 (36°C), *n* = 108 (40°C).

Next, we examined the responsiveness of HEK293-TRPV1 cells to a hypotonic stimulus at various temperatures. When the extracellular environment was changed from an isotonic (300 mOsm) to hypotonic (250 mOsm) solution at 24, 30, 36, and 40°C, [Ca^2+^]_i_ showed a slight decrease (Fig. 2). The amplitude of [Ca^2+^]_i_ decrease is small but significant. When osmolality was returned to 300 mOsm, [Ca^2+^]_i_ gradually recovered to the basal level (see, 8 min to 12 min in Fig. 2*A*). This suggests that some influx of Ca^2+^ occurs at a physiologically normal osmolality (∼300 mOsm) at 30–40°C and this entry is suppressed under hypotonic conditions. Of note, maximal sensitivity to the hypotonic stimulus was again observed at 36°C (Fig. 2*B*).

### Osmosensitive Responses Are Antagonized by TRPV1 Blockers

**Figure 3:**
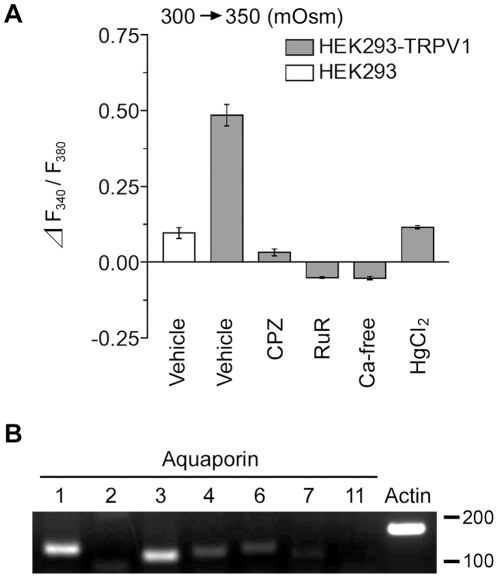
Pharmacological characterization of the osmotic response of HEK293-TRPV1 cells. (**A**) Summary of the change in fluorescence ratio during the perfusion of a hypertonic solution (350 mOsm) in the presence of the indicated reagents at 36°C in HEK293-TRPV1 (filled bars) and HEK293 (open bar) cells. Cells were preincubated for 15 min with an imaging buffer (300 mOsm) containing vehicle, 10 µM capsazepine (CPZ), 10 µM ruthenium red (RuR), Ca^2+^-free solution (Ca-free), or 1 mM HgCl_2_ (HgCl_2_), and then changed to a hypertonic solution containing the same reagents (350 mOsm). Data are differences between fluorescence ratios 2 min before and 4 min after the change of the solution. Values are the mean ± SEM. HEK293-TRPV1: *n* = 210 (Vehicle), *n* = 171 (CPZ), *n* = 174 (RuR), *n* = 137 (Ca-free), *n* = 156 (HgCl_2_). HEK293: *n* = 119 (Vehicle). (**B**) Expression of human *AQP1* and *AQP3* in HEK293-TRPV1 cells. Strong expression of the endogenous mRNAs of *AQP1* and *AQP3* was detected by RT-PCR. The mRNAs of *AQP4* and *AQP6* were also detected, though at low levels. Actin ß (Actin) was used as the endogenous control. Molecular size markers (base pair) are shown on the right.

Subsequently, we pharmacologically verified that the responses to osmotic stimuli observed in the HEK293-TRPV1 cells were mediated by TRPV1 channels. Application of capsazepine (10 µM), a specific inhibitor of TRPV1, and ruthenium red (10 µM), a nonspecific inhibitor of TRP channels, reduced the response to the basal level (Fig. 3*A*, CPZ and RuR), indicating that the osmosensitive responses of HEK293-TRPV1 cells were mediated by TRPV1. When hypertonic stimuli were applied with the Ca^2+^-free hypertonic solution, [Ca^2+^]_i_ did not change (Fig. 3*A*, Ca-free). Together, these results clearly indicate that the increase in [Ca^2+^]_i_ in the HEK293-TRPV1 cells under hypertonic conditions was mediated by TRPV1.

### Inhibition of Aquaporins Reduces the Osmotic Response of TRPV1

As cell membranes are highly permeable to water compared with ions, an increase or decrease in extracellular osmolality leads to the shrinkage or swelling of cells. Here, water channels, aquaporins (AQPs), are considered to contribute to the cellular shrinkage and swelling [17]. To test the possibility that the sensing of hypertonicity by TRPV1 is dependent on cell shrinkage through AQP activity, we examined the effect of HgCl_2_, which blocks AQPs and prevents a change in cell volume [18], [19]. Upon exposure to hypertonic solutions in the presence of 1 mM HgCl_2_ at 36°C, the response was significantly reduced (Fig. 3*A*, HgCl_2_). We also specified the expression of the AQP family in HEK293-TRPV1 cells by RT-PCR using primers for AQP members which are known to be expressed in the kidney (*AQP1*, *2*, *3*, *4*, *6*, *7*, and *11*; see [20]), because HEK293 cells are derived from human kidney. Among them, *AQP1* and *AQP3* were detected as main AQPs expressed in HEK293-TRPV1 cells (Fig. 3*B*).

### The Response Increases with a Rise in Osmolality

**Figure 4:**
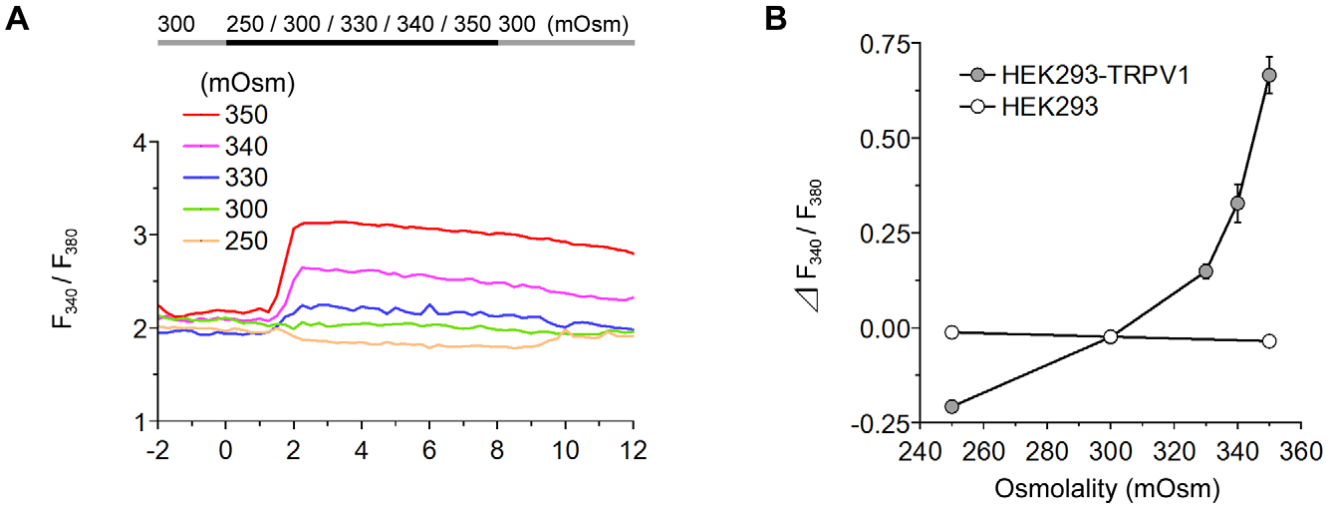
Response of TRPV1 to osmotic stimuli. (**A**) Representative single cell traces of the fluorescence ratio during the perfusion of various solutions differing in osmolality at 36°C. The line on the top indicates the timing of the change from 300 mOsm (gray) to the respective osmolality (black). (**B**) Summary of the change in the fluorescence ratio. Data are differences between fluorescence ratios 2 min before and 4 min after the change of the solution. Osmolality-dependent changes in the [Ca^2+^]_i_ were observed in HEK293-TRPV1 cells (filled circles), but not in HEK293 cells (open circles). Values are the mean ± SEM; when the SEM value was less than 0.04, the error bar is hidden behind the symbol. HEK293-TRPV1: *n* = 105 (250 mOsm), *n* = 110 (300 mOsm), *n* = 129 (330 mOsm), *n* = 109 (340 mOsm), *n* = 249 (350 mOsm). HEK293: *n* = 112 (250 mOsm), *n* = 121 (300 mOsm), *n* = 109 (350 mOsm).

Because HEK293-TRPV1 cells showed a maximal response at 36°C, we further investigated the response to the transfer from the physiological level (300 mOsm) to various osmotic levels at 36°C (Fig. 4*A*). The response of [Ca^2+^]_i_ upon the shift in extracellular osmolality is shown in Fig. 4*B*. The increase in [Ca^2+^]_i_ is clearly pronounced in a considerably hypertonic range (>330 mOsm).

### Hypertonic Response of TRPV1 Is Enhanced by Protons

**Figure 5:**
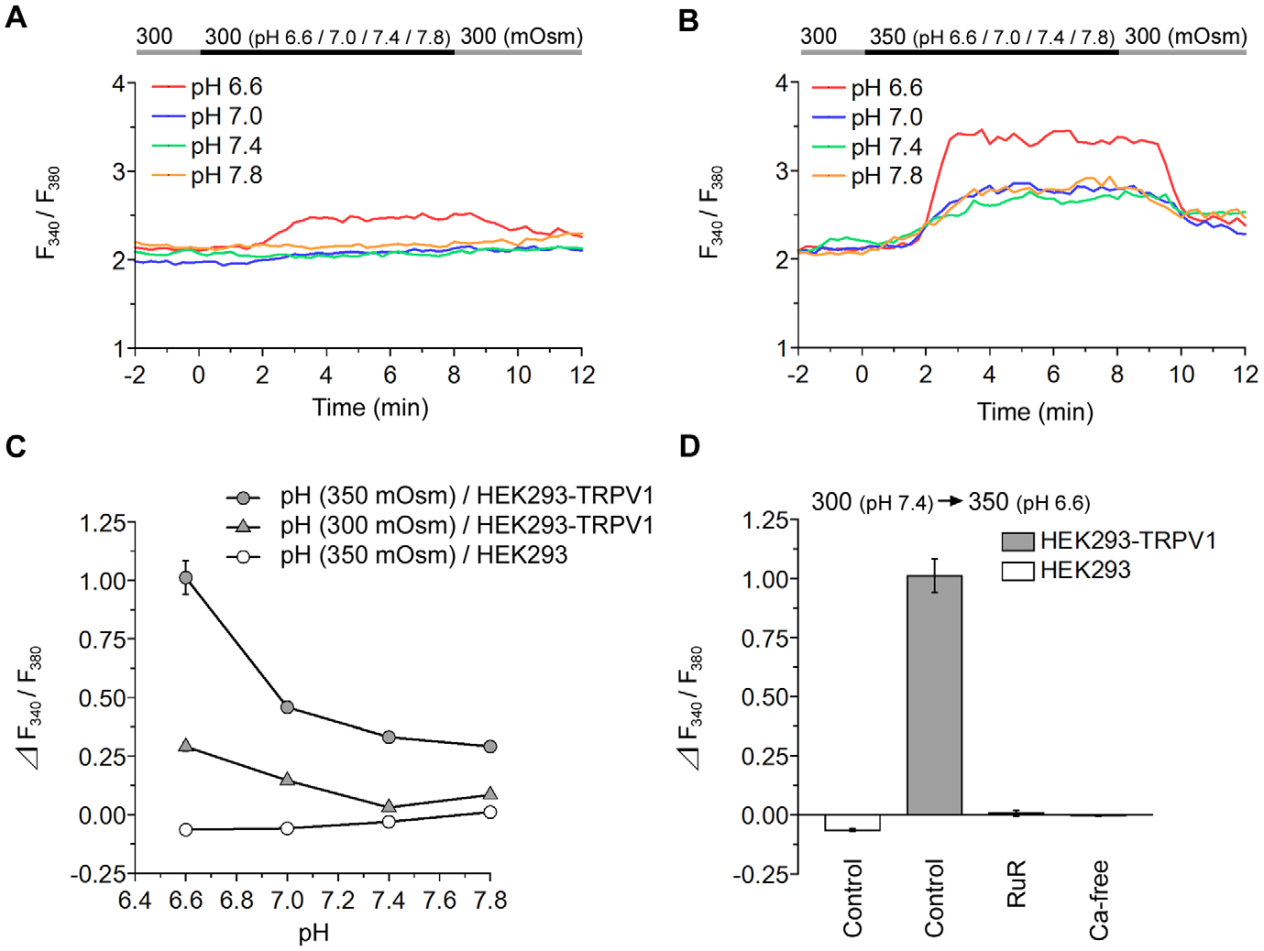
Hypertonic response of TRPV1 is potentiated by acidification. (**A**) Representative single cell traces of the fluorescence ratio in HEK293-TRPV1 cells during perfusion with isotonic solutions (300 mOsm) at various pH levels at 36°C. The line on the top indicates the timing of the change from pH 7.4 (gray) to the respective pH (black). (**B**) Representative single cell traces of the fluorescence ratio in HEK293-TRPV1 cells during the perfusion with hypertonic solutions (350 mOsm) at various pH levels at 36°C. The top line indicates the timing of the change from 300 mOsm at pH 7.4 (gray) to 350 mOsm at the respective pH (black). (**C**) Summary of the change in the fluorescence ratio. pH-dependent changes in the [Ca^2+^]_i_ were observed in HEK293-TRPV1 cells (filled triangles), but not HEK293 cells (opened circles). The response to pH in HEK293-TRPV1 cells (filled triangles) was observed as the pH decreased and was enhanced by hypertonic stimuli over the entire pH range (filled circles). (**D**) Summary of the change in the fluorescence ratio during the perfusion of a hypertonic acidic solution (350 mOsm, pH 6.6) in the presence of indicated reagents at 36°C in HEK293-TRPV1 (filled bars) and HEK293 (open bar) cells. Cells were preincubated for 15 min with an imaging buffer (300 mOsm) containing 20 µM ruthenium red (RuR) or Ca^2+^-free solution (Ca-free). Data are differences between fluorescence ratios 2 min before and 4 min after the change of the solution. Values are the mean ± SEM; when the SEM value was less than 0.07, the error bar is hidden behind the symbol. HEK293-TRPV1, isotonic stimuli: *n* = 144 (pH 6.6), *n* = 153 (pH 7.0), *n* = 151 (pH 7.4), *n* = 241 (pH 7.8). HEK293-TRPV1, hypertonic stimuli: *n* = 251 (pH 6.6), *n* = 150 (pH 7.0), *n* = 214 (pH 7.4), *n* = 148 (pH 7.8). HEK293, hypertonic stimuli: *n* = 156 (pH 6.6), *n* = 185 (pH 7.0), *n* = 149 (pH 7.4), *n* = 143 (pH 7.8). *n* = 176 (RuR), *n* = 214 (Ca-free).

Because protons are known to potentiate the response of TRPV1 to capsaicin [21] and heat [15], [21], we next examined the effect of acidification on the hypertonic response at 36°C (Fig. 5). As previously reported, a reduction in the extracellular pH itself induced an increase of [Ca^2+^]_i_ at 36°C from pH 6.6 in HEK293-TRPV1 cells, but not in control HEK293 cells (Fig. 5*A and C*). The [Ca^2+^]_i_ increase induced by pH 6.3 was far more large (ΔF_340_/F_360_ was ∼15.0; data not shown).

When a hypertonic solution (350 mOsm) at different pH levels (pH 7.8 to pH 6.6) was applied to HEK293-TRPV1 cells, [Ca^2+^]_i_ showed proportional increases according to the acidity (Fig. 5*B and C*). Studies with antagonists again indicated that the response was mediated by TRPV1 channels (Fig. 5*D*).

**Figure 6:**
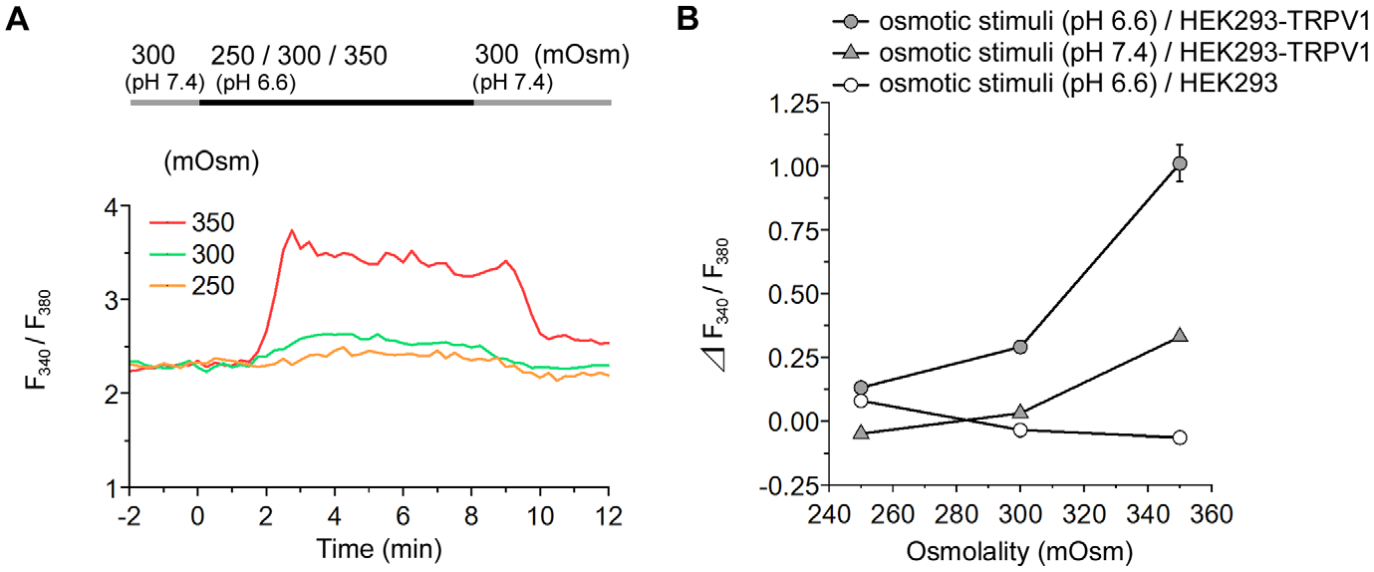
Osmotic response of TRPV1 is potentiated under acidic conditions. (**A**) Representative single cell traces of the fluorescence ratio during the perfusion of solutions with the respective osmolality at 36°C. The top line indicates the timing of the change from the control solution (pH 7.4, 300 mOsm; gray) to the acidic solution (pH 6.6) with the respective osmolality (black). (**B**) Summary of the change in the fluorescence ratio. Data are differences between fluorescence ratios 2 min before and 4 min after the change of the solution. The osmotic response of the [Ca^2+^]_i_ in HEK293-TRPV1 cells (filled circles) was enhanced by acidification over the entire osmolality range (compare with filled triangles). This was evident especially in the hypertonic range. Values are the mean ± SEM; when the SEM value was less than 0.07, the error bar is hidden behind the symbol. HEK293-TRPV1, pH 7.4: *n* = 138 (250 mOsm), *n* = 151 (300 mOsm), *n* = 214 (350 mOsm). HEK293-TRPV1, pH 6.6: *n* = 151 (250 mOsm), *n* = 144 (300 mOsm), *n* = 251 (350 mOsm). HEK293, pH 6.6: n = 129 (250 mOsm), *n* = 131 (300 mOsm), *n* = 156 (350 mOsm).

Importantly, the sensitivity of TRPV1 to an increase in osmolality was potentiated under acidic conditions (Fig. 6): compare pH 6.6 and pH 7.4 in HEK293-TRPV1 (Fig. 6*B*). These results indicate synergism between the osmo-sensitive and proton-sensitive responses. The small decrease in [Ca^2+^]_i_ by the hypotonic stimuli was changed to a small increase in [Ca^2+^]_i_ by the acidic pH shift (Fig. S2). This suggests that opening of TRPV1 by protons overcomes the closing of TRPV1 by hypotonicity under these conditions.

### Hypertonic Response of TRPV1 Is Enhanced by Capsaicin

**Figure 7:**
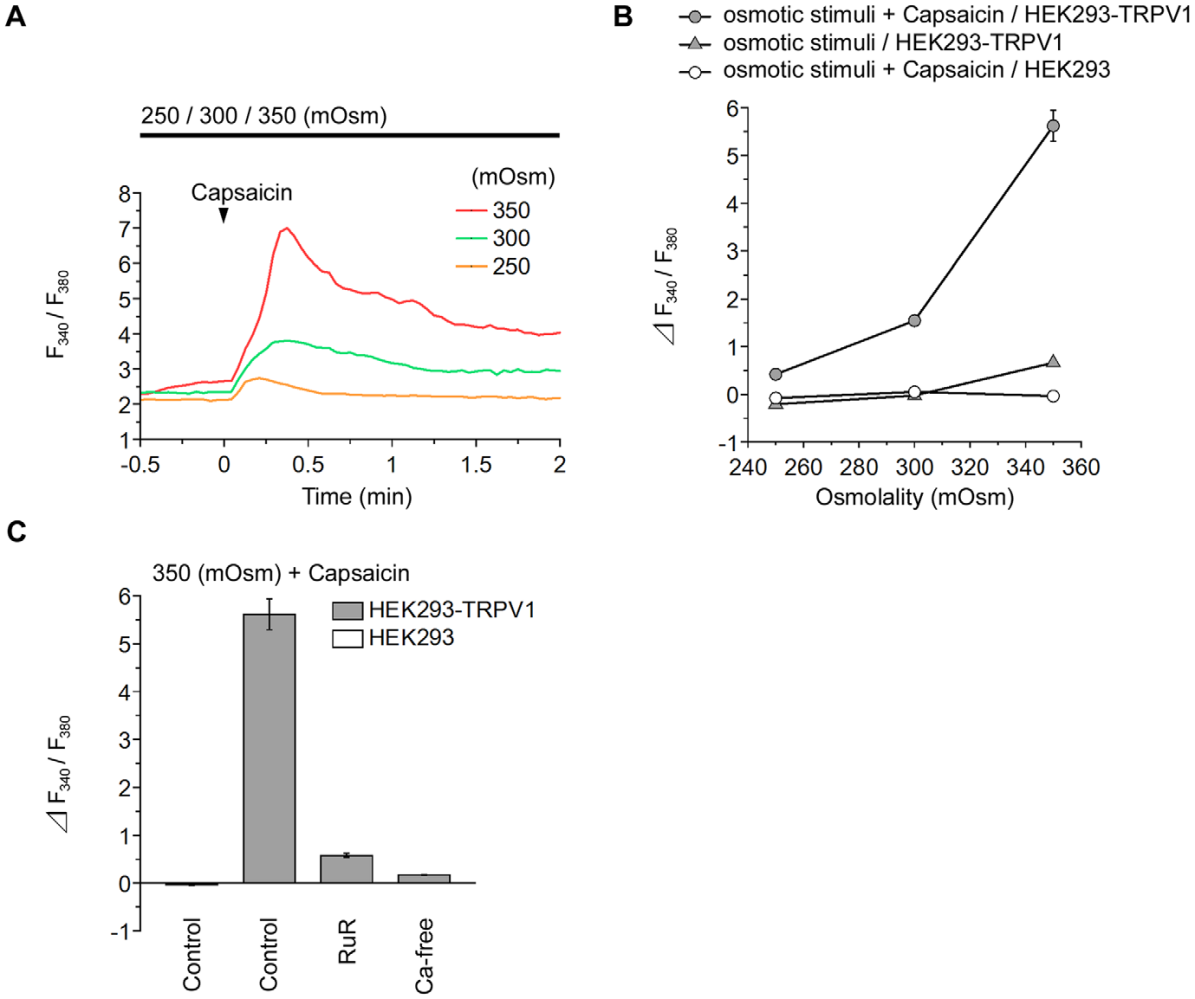
Synergistic effects of capsaicin and osmotic stimuli on TRPV1. (**A**) Representative single cell traces of the fluorescence ratio upon application of 1.5 nM capsaicin solutions (arrowhead) under various conditions of osmolality in HEK293-TRPV1 cells. The top black line indicates that the extracellular fluid with indicated osmolalities was applied before the capsaicin addition. Responses to capsaicin changed markedly with osmolality. (**B**) Summary of the change in the fluorescence ratio. Response of TRPV1 to hyperosmolality was markedly potentiated in the presence of capsaicin (compare filled circles with filled triangles). No response was observed in HEK293 cells (open circles). Data obtained at the peak was used to obtain the mean of the response. (**C**) Summary of the change in the fluorescence ratio during the perfusion of a hypertonic solution (350 mOsm) in the presence of capsaicin and indicated reagents at 36°C in HEK293-TRPV1 (filled bars) and HEK293 (open bar) cells. Cells were preincubated for 15 min with an imaging buffer (300 mOsm) containing 20 µM ruthenium red (RuR) or Ca^2+^-free solution (Ca-free). Data are differences between fluorescence ratios 2 min before and 4 min after the change of the solution. Values are the mean ± SEM; when the SEM value was less than 0.30, the error bar is hidden behind the symbol. HEK293-TRPV1: *n* = 305 (250 mOsm), *n* = 390 (300 mOsm), *n* = 220 (350 mOsm), *n* = 136 (RuR), *n* = 112 (Ca-free). HEK293: *n* = 108 (250 mOsm), *n* = 113 (300 mOsm), *n* = 114 (350 mOsm).

Finally, we examined the relationship between hypertonicity and capsaicin as activating stimuli for TRPV1 at 36°C. We found that the response to capsaicin was potentiated as the osmolality increased, and *vice versa* (Fig. 7*A*). The osmotic response was markedly enhanced by costimulation with capsaicin (Fig. 7*B*; compare osmotic stimuli capsaicin with osmotic stimuli in HEK293-TRPV1). Studies with antagonists demonstrated that the response was mediated by TRPV1 channels (Fig. 7*C*).

## Discussion

The present study demonstrates that the full-length form of the TRPV1 channel responds to hypertonic stimuli in a temperature-dependent manner. Rat TRPV1 showed peak sensitivity at around the body temperature (36°C) of mammals. In this study, we also demonstrated that the osmosensitivity of TRPV1 at 36°C is further enhanced by another activating stimulus, such as protons (pH) or capsaicin. Inhibition of the water channels significantly decreased the hypertonic response of TRPV1. This suggests that a reduction in cell volume or the tension of the plasma membrane is important for the osmosensitive gating mechanism of TRPV1, as in TRPV4 [22].

### Sensitivity of TRPV1 to Hypertonic Stimuli

To our knowledge, only one protein, stretch-inhibitable cation channel (SIC), has been shown to have hypertonicity-sensitive properties in a heterologous expression system [23]. However, it was later found that SIC was an artificial chimeric channel composed of partial fragments of TRPV1 and TRPV4: the core is derived from an N- and C-terminal-deleted TRPV1 containing all of the membrane-spanning regions, and the C-terminal domain is derived from TRPV4 [24]. However, the response of TRPV1 itself to hypertonic stimuli has not been demonstrated with heterologous expression systems so far, despite the intense efforts of several groups. Our finding that the osmosensitive response of TRPV1 is very small at room temperature (24°C) may explain why it has not been identified previously, because researchers used to employ room temperature for the experiments. Further study will be required to confirm whether the peak sensitivity of TRPV1 to hypertonic stimuli occurs at the particular body temperature of the individual animal species.

Liu et al. recently demonstrated that osmolality potentiates the response to capsaicin of trigeminal sensory neurons [25]. Moreover, the responsiveness of TRPV1 to protons and chemical agonists including capsaicin was markedly enhanced above room temperature in a heterologous expression system like ours [21], [26]. These results are in line with our findings that osmosensitive activity of TRPV1 is enhanced by not only temperature but also protons and capsaicin. Here it should be noted that the [Ca^2+^]_i_ increase induced by hyperosmolality (350 mOsm) at 36°C (see Fig. 1*D*) is comparable to that induced by capsaicin (1.5 nM) at 36°C (see Fig. 7*B*; 300 mOsm). All these findings should be further confirmed by using endogenous cells expressing TRPV1.

### Physiological Role of the Osmosensitivity of TRPV1

Channels mediating osmosensory transduction in the magnocellular neurons in the SON are reportedly permeable to calcium and can be blocked by the extracellular application of gadolinium (a non-selective cation channel inhibitor) or ruthenium red [8], [27], [28]. The response of osmosensitive neurons in the OVLT and SON to hypertonic stimulation was abolished in brain slices obtained from *TRPV1*-KO mice [7], [8]. These findings suggested that a *TRPV1* gene product plays an important role in osmosensory transduction. Our present study clearly demonstrates that the full-length form of TRPV1 is sensitive to extracellular hypertonic stimuli at around the normal core body temperature of mammals. Although it is not yet clear whether the sensitivity is sufficient to detect the osmotic change within the physiological range *in vivo*, our findings would be an important contribution to understanding the mechanism for the osmotic homeostasis of body fluids. The discrepancies above noted among experimental results with *TRPV1* or *TRPV4* gene-KO animals might be attributable to differences in the protocols used for osmotic stimulation. Further research employing more solid experimental techniques might be required to clarify the physiological role of TRPV1 and TRPV4 in body-fluid regulation.

### Pathological Meaning of Integration of Multiple Stimuli in TRPV1

Our finding that TRPV1 is synergistically regulated by distinct stimuli for activation may contribute to our pathological understanding of several diseases. Because acidification potentiated the response of TRPV1 to hypertonic stimuli, it is presumable that control of body-fluid osmolality is affected by acidosis. Diabetic acidosis is an acute metabolic complication of diabetes, and dry mouth and excessive drinking are major symptoms of diabetes [29]. Because TRPV1 underlies thirst responses in mammals, the drinking response induced by a hypertonic state is considered to be enhanced by acidosis in diabetic patients, as a result of the integration of hypertonic and acidic stimuli in TRPV1.

Another physiological situation involving the integration of distinct stimuli in TRPV1 may occur in pain sensation. It has been postulated that TRPV1 senses a reduction of pH in tissues caused by infection, inflammation, or ischemia, which produces pain in mammals [15], [21]. Presumably, osmolality is also increased in damaged tissues [30], [31]. It is known that the injection of a hypertonic solution into skin or muscle causes pain [32]. TRPV1 is thus considered an integrator of the physicochemical noxious signals derived from inflammatory injuries. Taken altogether, our findings provide a novel view of TRPV1 that this sensor integrates multiple combinations of distinct physiological stimuli.

## Materials and Methods

### TRPV1-Expressing Cells

To explore the properties of the full-length form of TRPV1, HEK293-TRPV1 cells were used [33]. The cells were plated on glass cover slips (CS-12R, Warner) and cultured in Dulbecco's Modified Eagle Medium (DMEM, Nissui Pharmaceutical) containing 10% fetal calf serum (FCS, Invitrogen) under 5% CO_2_ at 37°C for at least 24 h before imaging. For the detection of TRPV1 expression, cells were fixed with 2% (v/v) neutralized formalin (Wako) for 15 min and washed three times with PBS containing 0.5% Triton X-100 (Nacalai Tesque). They were then incubated with anti-TRPV1 antibody (1∶1200; RA14113, Neuromics) in PBS containing 10% normal goat serum (Cosmo Bio) for 1.5 h, and subsequently with the secondary antibody, Alexa488-conjugated anti-rabbit IgG antibody (1∶1200, Invitrogen), for 40 min.

### Reverse Transcription (RT)-PCR

Total RNA was isolated from HEK293-TRPV1 cells by the Trizol (Invitrogen) method. Oligo(dT)-primed cDNA was synthesized with the SuperScript III reverse transcriptase (Invitrogen). RT-PCR was performed for 40 cycles, with each cycle consisting of 94°C for 30 sec, 60°C for 30 sec, and 72°C for 30 sec with the primer sets to detect expression of rat TRPV1 (TRPV1(5′) and TRPV1(3′)), human aquaporins (AQPs), and Actin ß (Actin) mRNAs. The sequences of the primers were shown in Table S1. The PCR products were sequenced for confirmation.

### Calcium Imaging

The activation of the TRPV1 channels was detected by ratiometric calcium imaging. Imaging buffers differing in osmolality were made by adding an appropriate amount of mannitol to a basal hypotonic solution (250 mOsm) containing (in mM) 110 NaCl, 2.5 KCl, 2.5 CaCl_2_, 1 MgCl_2_, 10 HEPES, 5 glucose, and 5 NaOH, pH 7.3. The pH (6.6–7.8) was adjusted with NaOH using a pH meter (HM-16S, TOA Electronics). The osmolality was determined with an osmometer (One-ten, Fiske associates). Cells were loaded with 4 µM Fura-2 acetoxymethyl ester (Molecular Probes) and 0.01% Pluronic F-127 (Sigma) for 30 min at 37°C beforehand.

During the experiments, the cells were perfused with the imaging buffer at 0.5 ml/min. Before the start of the imaging, cells were placed in the chamber for at least 15 min of perfusion with the isotonic imaging buffer (300 mOsm) to stabilize the basal level of the intracellular Ca^2+^ concentration ([Ca^2+^]_i_). The perfusate was changed to a test imaging buffer of different osmolality and/or pH using two peristaltic pumps controlled with a controller (Gradicon III, Atto). The ambient temperature of the cells was maintained with a chamber heater and in-line heater adjusted with a dual heater controller (TC-344B, Warner). All the experiments were performed at temperatures below the activation threshold of TRPV1 (>43°C).

For the experiments to examine the dependency on osmolality of the response to capsaicin, the perfusion was stopped and the remaining fluid in the chamber was replaced with 200 µl of solutions at various osmotic levels. After the Ca^2+^ imaging was started, capsaicin was added to the chamber at 1.5 nM (final concentration).

The fluorescence at excitation wavelengths of 340 and 380 nm was measured using a microscope equipped with a cooled charge coupled device (cooled CCD) camera (ORCA-ER, Hamamatsu Photonics). Data were collected every 15 sec, except for experiments on the response to capsaicin (2.5 sec), and analyzed using image-analysis software (AQUACOSMOS version 2.5, Hamamatsu Photonics). Elevated relative calcium concentrations are indicated by an increased ratio of Fura-2 emission at 340 versus 380 nm (F_340_/F_380_). The change in fluorescence ratio is the difference between the F_340_/F_380_ values 2 min before and 4 min after the change of the extracellular solution.

For the pharmacological experiments, 10 µM capsazepine (Sigma), 10 or 20 µM ruthenium red (Sigma), and 1 mM HgCl_2_ (Nacalai Tesque) were added to the perfusate, respectively. For the Ca^2+^-free perfusate, buffers without CaCl_2_ were used. All the solutions used for the pharmacological experiments contained 0.1% EtOH.

## Supporting Information

Figure S1 ***TRPV1*** **is expressed in HEK293-TRPV1 but not in HEK293 cells.** Schematic drawing of rat *TRPV1* mRNA (top). The exon structure (exon1 to exon16) is shown with vertical lines. Arrowheads indicate translational start and stop codons. Horizontal lines underneath illustrate mRNA regions amplified with primer sets for 5′ and 3′, respectively. Analysis of the PCR products (bottom). Strong expression of the mRNA of *TRPV1* was detected by RT-PCR in HEK293-TRPV1 cells, but not HEK293 cells. Molecular size markers (base pairs) are shown on the right.
(TIF)

Figure S2 **Hypotonic response of TRPV1 is not potentiated by acidification.** **(A)** Representative single cell traces of the fluorescence ratio during the perfusion with hypotonic solutions (250 mOsm) of various pH at 36°C. The top line indicates the timing of the change from 300 mOsm, pH 7.4 (gray) to 250 mOsm of various pH (black). **(B)** Summary of the change in the fluorescence ratio during the perfusion with the hypotonic solution at various pH values in HEK293-TRPV1 (filled circles) and HEK293 (open circles) cells. Data are differences between fluorescence ratios 2 min before and 4 min after the change of the solution. Values are the mean ± SEM; when the SEM value was less than 0.07, the error bar is hidden behind the symbol. HEK293-TRPV1: *n* = 151 (pH 6.6), *n* = 178 (pH 7.0), *n* = 195 (pH 7.4). HEK293: *n* = 129 (pH 6.6), *n* = 125 (pH 7.0), *n* = 126 (pH 7.4).
(TIF)

Table S1 Primer sequences for RT-PCR.
(DOC)

## References

[1] Andersson B (1977). Regulation of body fluids.. Annu Rev Physiol.

[2] Andersson B (1978). Regulation of water intake.. Physiol Rev.

[3] Arieff AI (1993). Management of hyponatraemia.. BMJ.

[4] Ayus JC, Armstrong DL, Arieff AI (1996). Effects of hypernatraemia in the central nervous system and its therapy in rats and rabbits.. J Physiol.

[5] Stiefel D, Petzold A (2007). H_2_O coma.. Neurocritical Care.

[6] Bourque CW (2008). Central mechanisms of osmosensation and systemic osmoregulation.. Nat Rev Neurosci.

[7] Sharif Naeini R, Witty MF, Séguéla P, Bourque CW (2006). An N-terminal variant of Trpv1 channel is required for osmosensor transduction.. Nat Neurosci.

[8] Ciura S, Bourque CW (2006). Transient receptor potential vanilloid 1 is required for intrinsic osmoreception in organum vasculosum lamina terminalis neurons and for normal thirst responses to systemic hyperosmolality.. J Neurosci.

[9] Taylor AC, McCarthy JJ, Stocker SD (2008). Mice lacking the transient receptor vanilloid potential 1 channel display normal thirst responses and central Fos activation to hypernatremia.. Am J Physiol Regul Integr Comp Physiol.

[10] Liedtke W, Choe Y, Martí-Renom MA, Bell AM, Denis CS (2000). Vanilloid receptor-related osmotically activated channel (VR-OAC), a candidate vertevrate osmoreceptor.. Cell.

[11] Strotmann R, Harteneck C, Nunnenmacher K, Schultz G, Plant TD (2000). OTRPC4, a nonselective cation channel that confers sensitivity to extracellular osmolarity.. Nat Cell Biol.

[12] Wissenbach U, Bödding M, Freichel M, Flockerzi V (2000). Trp12, a novel Trp related protein form kidney.. FEBS Lett.

[13] Liedtke W, Friedman JM (2003). Abnormal osmotic regulation in *trpv4^-/-^* mice.. Proc Natl Acad Sci U S A.

[14] Mizuno A, Matsumoto N, Imai M, Suzuki M (2003). Impaired osmotic sensation in mice lacking TRPV4.. Am J Physiol Cell Physiol.

[15] Caterina MJ, Schumacher MA, Tominaga M, Rosen TA, Levine JD (1997). The capsaicin receptor: a heat-activated ion channel in the pain pathway.. Nature.

[16] Shuttleworth TJ, Thompson JL (1991). Effect of temperature on receptor-activated changes in [Ca^2+^]*_i_* and their determination using fluorescent probes.. J Biol Chem.

[17] Borgnia M, Nielsen S, Engel A, Agre P (1999). Cellular and molecular biology of the aquaporin water channels.. Annu Rev Biochem.

[18] Ishibashi K, Sasaki S, Fushimi K, Uchida S, Kuwahara M (1994). Molecular cloning and expression of a member of the aquaporin family with permeability to glycerol and urea in addition to water expressed at the vasolateral membrane of kidney collecting duct cells.. Proc Natl Acad Sci U S A.

[19] Jung JS, Bhat RV, Preston GM, Guggino WB, Baraban JM (1994). Molecular characterization of an aquaporin cDNA from brain: Candidate osmoreceptor and regulator of water balance.. Proc Natl Acad Sci U S A.

[20] Nielsen S, Frøkiær J, Marples D, Kwon TH, Agre P (2002). Aquaporins in the kidney: from molecules to medicine.. Physiol Rev.

[21] Tominaga M, Caterina MJ, Malmberg AB, Rosen TA, Gilbert H (1998). The cloned capsaicin receptor integrates multiple pain-producing stimuli.. Neuron.

[22] Liu X, Bandyopadhyay B, Nakamoto T, Singh B, Liedtke W (2006). A role for AQP5 in activation of TRPV4 by hypotonicity.. J Biol Chem.

[23] Suzuki M, Sato J, Kutsuwada K, Ooki G, Imai M (1999). Cloning of a stretch-inhibitable nonselective cation channel.. J Biol Chem.

[24] Xue Q, Yu Y, Trilk SL, Jong BE, Schumacher MA (2001). The genomic organization of the gene encoding the vanilloid receptor: evidence for multiple splice variants.. Genomics.

[25] Liu L, Chen L, Liedtke W, Simon SA (2007). Changes in osmolality sensitize the response to capsaicin in trigeminal sensory neurons.. J Neurophysiol.

[26] Sprague J, Harrison C, Rowbotham DJ, Smart D, Lambert DG (2001). Temperature-dependent activation of recombinant rat vanilloid VR1 receptors expressed in HEK293 cells by capsaicin and anandamide.. Eur J Pharmacol.

[27] Oliet SHR, Bourque CW (1996). Gadolinium uncouples mechanical detection and osmoreceptor potential in supraoptic neurons.. Neuron.

[28] Zhang Z, Bourque CW (2006). Calcium permeability and flux through osmosensory transduction channels of isolated rat supraoptic nucleus neurons.. Eur J Neurosci.

[29] Kitabchi AE, Nyenwe EA (2006). Hyperglycemic crises in diabetes mellitus: diabetic ketoacidosis and hyperglycemic hyperosmolar state.. Endocrinol Metab Clin N Am.

[30] Spector WG (1956). The mediation of altered capillary permeability in acute inflammation.. J Pathol Bacteriol.

[31] Vakili C, Ruiz-Ortiz F, Burke JF (1970). Chemical and osmolar changes of interstitial fluid in acute inflammatory states.. Surg Forum.

[32] Hamamoto DT, Forkey MW, Davis WL, Kajander KC, Simone DA (2000). The role of pH and osmolarity in evoking the acetic acid-induced wiping response in a model of nociception in fogs.. Brain Res.

[33] Güler AD, Lee H, Iida T, Shimizu I, Tominaga M (2002). Heat-evoked activation of the ion channel, TRPV4.. J Neurosci.

