# Supplementary figures and images for: Osmosensitivity of Transient Receptor Potential Vanilloid 1 Is Synergistically Enhanced by Distinct Activating Stimuli Such as Temperature and Protons
Source: PLoS One. 2011 Jul 14;6(7):e22246. doi: 10.1371/journal.pone.0022246 (PMC3136519; doi:10.1371/journal.pone.0022246)

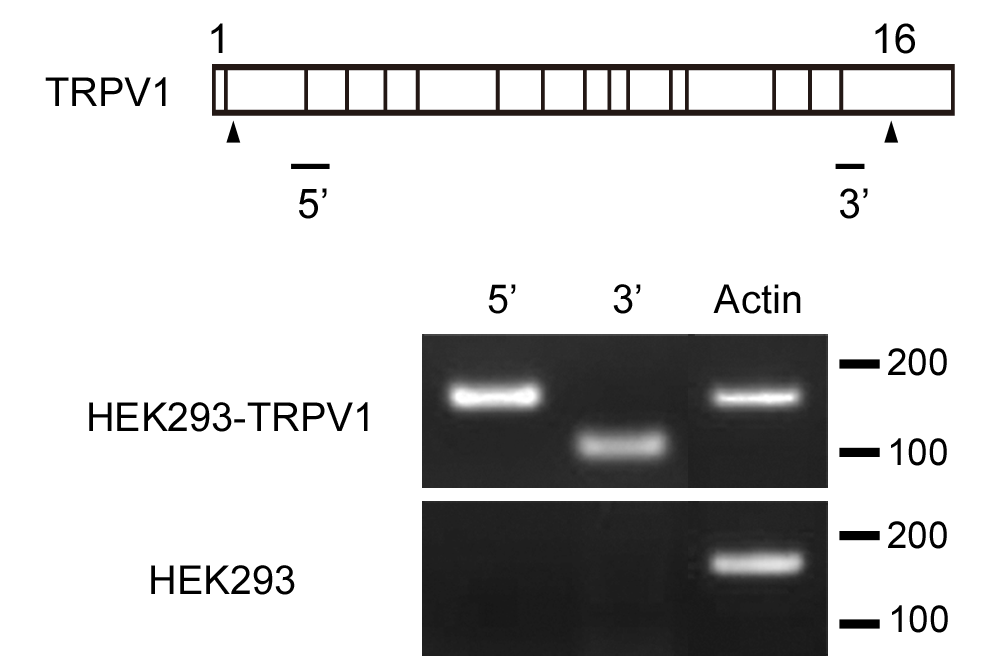

Supplement: Figure S1 — TRPV1 is expressed in HEK293-TRPV1 but not in HEK293 cells. Schematic drawing of rat TRPV1 mRNA (top). The exon structure (exon1 to exon16) is shown with vertical lines. Arrowheads indicate translational start and stop codons. Horizontal lines underneath illustrate mRNA regions amplified with primer sets for 5′ and 3′, respectively. Analysis of the PCR products (bottom). Strong expression of the mRNA of TRPV1 was detected by RT-PCR in HEK293-TRPV1 cells, but not HEK293 cells. Molecular size markers (base pairs) are shown on the right. (TIF) [file pone.0022246.s001.tif]

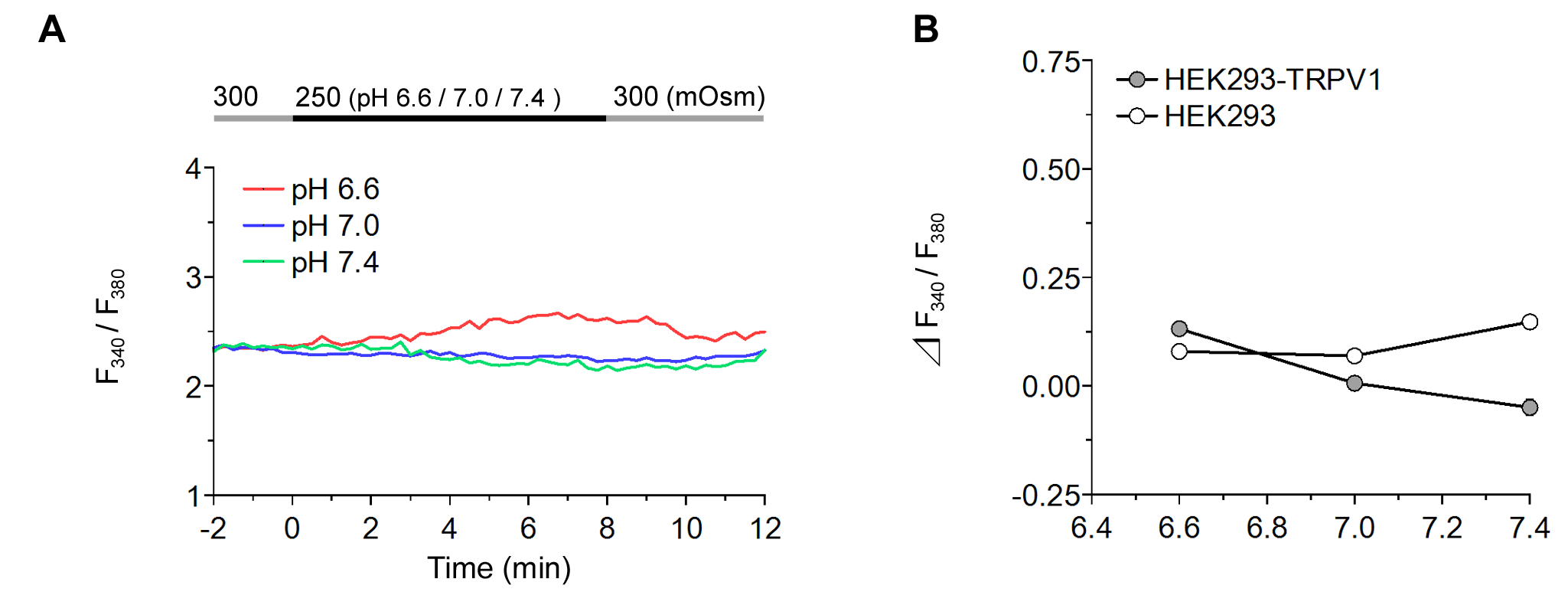

Supplement: Figure S2 — Hypotonic response of TRPV1 is not potentiated by acidification. (A) Representative single cell traces of the fluorescence ratio during the perfusion with hypotonic solutions (250 mOsm) of various pH at 36°C. The top line indicates the timing of the change from 300 mOsm, pH 7.4 (gray) to 250 mOsm of various pH (black). (B) Summary of the change in the fluorescence ratio during the perfusion with the hypotonic solution at various pH values in HEK293-TRPV1 (filled circles) and HEK293 (open circles) cells. Data are differences between fluorescence ratios 2 min before and 4 min after the change of the solution. Values are the mean ± SEM; when the SEM value was less than 0.07, the error bar is hidden behind the symbol. HEK293-TRPV1: n = 151 (pH 6.6), n = 178 (pH 7.0), n = 195 (pH 7.4). HEK293: n = 129 (pH 6.6), n = 125 (pH 7.0), n = 126 (pH 7.4). (TIF) [file pone.0022246.s002.tif]
