# Supplementary material for: Osmosensitivity of Transient Receptor Potential Vanilloid 1 Is Synergistically Enhanced by Distinct Activating Stimuli Such as Temperature and Protons
Source: PLoS One. 2011 Jul 14;6(7):e22246. doi: 10.1371/journal.pone.0022246 (PMC3136519; doi:10.1371/journal.pone.0022246)
Supplement: Table S1 — Primer sequences for RT-PCR. (DOC) [file pone.0022246.s003.doc]

**Table S1. Primer sequences for RT-PCR**

| **Primer name** | **Sequence (5’→3’)** |
| --- | --- |
| TRPV1(5’)-F | GCTTCCTGCCCTATCATCAC |
| TRPV1(5’)-R | CCACAGCATCGAAGATGCTCCTGC |
| TRPV1(3’)-F | ACTGTGAGGGCGTCAAGC |
| TRPV1(3’)-R | TCCCTCAGAAGGGGAACC |
| AQP1-F | GCCTCTCTGTAGCCCTTGG |
| AQP1-R | CACCCAGAAAATCCAGTGGT |
| AQP2-F | CGGCTGCTCTATGAATCCTG |
| AQP2-R | AGGGGTCCGATCCAGAAG |
| AQP3-F | ATCGCTGGCCAGGTCTCT |
| AQP3-R | CGTCTGTGCCAGGGTGTA |
| AQP4-F | CTTCTACATCGCAGCCCAGT |
| AQP4-R | CGGTAAGATTTCCATGAACCA |
| AQP6-F | ATTGGGATCTCTGTGGCACT |
| AQP6-R | CCCAGAAGACCCAGTGGAC |
| AQP7-F | TTGCCACCTACCTTCCTGAT |
| AQP7-R | TTGTTCTCCTGGTCCGTGA |
| AQP11-F | CAGGAAGTCCGAACCAAGC |
| AQP11-R | GAAAGTGCCAAAGCTGGATTA |
| Actin-F | CATCCGCAAAGACCTGTACGCCAAC |
| Actin-R | ATGGAGCCGCCGATCCACA |
